# Antimicrobial Consumption and Prescribing Patterns in Hospitalized Children During and After the COVID-19 Pandemic

**DOI:** 10.3390/pathogens15080841

**Published:** 2026-08-12

**Authors:** Sandra Prgomet, Danica Boban, Zvonimir Boban, Nataša Boban

**Affiliations:** 1 Department of Pediatrics, University Hospital Centre Split, 21000 Split, Croatia; 2 Department of Anatomy, Histology and Embryology, University of Split School of Medicine, 21000 Split, Croatia; 3 Department of Medical Physics and Biophysics, University of Split School of Medicine, 21000 Split, Croatia; 4 Department of Hospital Infections and Clinical Epidemiology, University Hospital Split, 21000 Split, Croatia; 5 Department of Public Health, University of Split School of Medicine, 21000 Split, Croatia

**Keywords:** antimicrobial stewardship, antibiotic consumption, pediatric hospitalization, AWaRe classification, days of therapy, length of therapy, days of antibiotic spectrum coverage, ceftriaxone, watch antibiotics, broad-spectrum antibiotics

## Abstract

The COVID-19 pandemic shifted pediatric infection epidemiology from bacterial toward viral predominance as public health measures relaxed. To determine how hospital antibiotic prescribing adapted, antimicrobial consumption was characterized and linked to underlying pathogen type. Antibiotic use was retrospectively analyzed among 567 children admitted with a positive microbiological finding to the pediatric ward of a tertiary hospital in Split, Croatia, during the first quarters of 2021–2024. Consumption was quantified using Days of Therapy (DOT), Length of Therapy (LOT), and Days of Antibiotic Spectrum Coverage (DASC) per 1000 patient-days (PD). Antibiotics were grouped by the WHO Access/Watch/Reserve (AWaRe) classification and linked to discharge diagnosis and pathogen type. The proportion of children receiving antibiotics rose from 60.5% (26/43) in 2021 to 71.4% (130/182) in 2024, with the cohort shifting toward younger infants and virally driven diagnoses. DOT, DASC, and DOT/LOT peaked in 2022 (DOT 2164/1000 PD, DOT/LOT 2.2) before settling near 1400/1000 PD, with mean per-day spectrum (DASC/DOT) declining modestly (7.7 to 6.8). Watch-group antibiotics dominated throughout (~80%), with Access contribution of ~20% and Reserve antibiotic use negligible. Ceftriaxone usage rose fivefold (150 to 755 DOT/1000 PD) to become the leading agent. Ceftriaxone and azithromycin were administered more often to children with viral than bacterial infections (respiratory syncytial virus being most common), whereas vancomycin and meropenem were tied to bacterial isolates. These findings identify a high-yield stewardship target and underscore the value of multi-metric, pathogen-aware surveillance.

## 1. Introduction

Antimicrobial resistance (AMR) poses a great risk to global public health [1,2]. The most comprehensive estimates to date attribute an enormous burden to bacterial AMR, with 1.14 million deaths in 2021 attributable to drug-resistant infections and roughly 4.71 million associated with them, with children disproportionately affected [1]. Because the principal modifiable driver of resistance is the misuse and overuse of antimicrobials, optimizing antibiotic prescribing, particularly in hospitals where broad-spectrum agents are concentrated, is central for AMR containment strategies.

A substantial proportion of childhood hospitalizations are due to respiratory infections, the overwhelming majority of which are viral and therefore unresponsive to antibiotic therapy. Nevertheless, antibiotics are frequently administered to children with viral illness, most often out of concern for possible bacterial co-infection. Contemporary studies show that approximately one third of children with RSV and 60% of children with influenza were prescribed antibiotics even though they did not have a confirmed bacterial co-infection [3,4]. Furthermore, the WHO reports that approximately 75% of COVID-19 patients have been treated with antibiotics, although only 8% had bacterial co-infections [5]. Such redundant prescribing carries risk of antibiotic-associated adverse events and a heightened future risk of resistant infection, as well as population-level consequences for AMR.

To standardize the rational use of antibiotics, the World Health Organization (WHO) introduced the Access, Watch, and Reserve (AWaRe) classification, which groups antibiotics by their resistance-selection potential [6]. Access agents are first-line therapies for common infections with low resistance potential, Watch agents carry a higher resistance risk and should be reserved for specific indications, and Reserve agents are last-line options for multidrug-resistant pathogens. The WHO has set the target that Access antibiotics should constitute at least 60% of consumption, rising toward 70% of prescriptions globally by 2030. In practice, many hospital settings fall well short of this benchmark, with a persistent overreliance on Watch-group agents and underuse of narrow-spectrum Access drugs [7,8].

Quantifying consumption requires standardized metrics. Days of Therapy (DOT) and Length of Therapy (LOT), normalized to patient-days (PD), are the most widely used measures of antibiotic exposure but capture only the quantity of use, not its breadth. To address this limitation, the Days of Antibiotic Spectrum Coverage (DASC) metric was developed [9], weighing each antibiotic-day by an antibiotic spectrum coverage score so that broader-spectrum and combination regimens yield proportionally higher values, overcoming the tendency of DOT to overlook stewardship efforts that favor narrow-spectrum agents. The derived DASC/DOT ratio reflects the average spectrum coverage per therapy-day. Together, these metrics allow consumption to be evaluated in both quantitative and qualitative terms [9,10].

The COVID-19 pandemic significantly altered both the epidemiology of pediatric infection and the conditions of antibiotic prescribing. Non-pharmaceutical interventions suppressed the circulation of common respiratory and enteric viruses, and as mitigation measures were lifted, respiratory pathogens and care-seeking patterns moved back toward their pre-pandemic seasonality, frequently with out-of-season resurgences. At the same time, antibiotic use in patients with COVID-19 was high and broad despite a low incidence of bacterial co-infection [3,5].

Our recent study analyzing the transition from the pandemic to post-pandemic period in hospitalized children showed a roughly fourfold rise in admissions, a fall in mean patient age driven by an influx of infants, and a shift from bacterial to viral predominance, with RSV and influenza overtaking SARS-CoV-2 and rotavirus as the easing of restrictions allowed viral resurgence [11]. Building on that work, we quantified antimicrobial consumption using DOT, LOT, and DASC, classified antibiotics according to the WHO AWaRe framework, and linked each prescription to the discharge diagnosis and to whether the patient’s identified pathogen was viral or bacterial. The aim was to determine whether prescribing adapted to the documented shift toward viral infection, or persisted independently of it.

## 2. Materials and Methods

### 2.1. Study Design and Population

This was a retrospective, single-center study conducted at the Pediatric Clinic of University Hospital Split, a tertiary care center in Split, Croatia. The study population comprised all children aged 0–18 years with a positive microbiological finding who were admitted during the first quarter (1 January–31 March) of each of the years 2021 to 2024. These four first-quarter periods were chosen to capture the season of peak respiratory infection activity while spanning the period of strict non-pharmaceutical interventions in 2021 through their progressive relaxation and the post-pandemic period of 2022–2024. A total of 567 children met these inclusion criteria and constituted the base cohort. The present analysis focuses on the subset of these children who received at least one antibiotic during admission (*n* = 382).

### 2.2. Data Collection

The study was conducted in accordance with the Declaration of Helsinki and was approved by the Ethics Committee of University Hospital Split (No. 2181-147/01-06/LJ.Z.-25-02) on 30 April 2025. The dataset was deidentified and only aggregate statistics were calculated and analyzed.

Data were retrieved from the hospital’s electronic health record system and anonymized prior to analysis to protect patient confidentiality. For each patient, demographic characteristics (age and sex), length of hospital stay (LOS), discharge diagnosis (all infection types documented in a patient’s discharge diagnosis were included in the dataset), microbiological findings, and the complete inpatient antimicrobial administration record were extracted. The antibiotic data comprised the identity of each systemic antimicrobial agent administered and the calendar days on which it was given, allowing day-level reconstruction of each patient’s therapy. Only systemic administration routes (intravenous and oral) were recorded, with non-systemic routes (e.g., topical) excluded, consistent with prior DASC/DOT methodology. Microbiological isolates were identified using the diagnostic techniques routinely employed at the center, including bacterial cultures (coproculture, urinoculture, and hemoculture with subsequent pathogen identification and antibiotic susceptibility testing), rapid immunochromatographic antigen tests, multiplex PCR of nasopharyngeal swabs for respiratory pathogens, real-time reverse-transcription PCR for SARS-CoV-2, and serology. See the Section 2 of our previous study for more details on diagnostic testing [11].

### 2.3. Antibiotic Consumption Metrics

Antibiotic consumption was quantified using three complementary day-level metrics: Length of Therapy (LOT), Days of Therapy (DOT), and Days of Antibiotic Spectrum Coverage (DASC). LOT was defined as the number of days on which a patient received at least one systemic antibiotic, irrespective of the number of agents administered. DOT was defined as the sum of the days of administration of each individual agent, so that a day on which a patient received more than one antibiotic contributed more than one DOT. DASC was calculated by weighting each antibiotic-day by a standardized spectrum score reflecting the breadth of antibacterial coverage of the agent administered and summing these scores across the therapy. To allow comparison across periods with differing numbers of admissions and bed occupancy, all three metrics were normalized to total patient-days (PD) and expressed per 1000 PD, where patient-days were calculated as the sum of the lengths of stay of the relevant patients. Two derived ratios were also computed: DOT/LOT, representing the mean number of antibiotics administered concurrently per therapy-day, and DASC/DOT, representing the mean spectrum breadth per antibiotic-day. Each antibiotic was also assigned to one of the three categories of the World Health Organization Access, Watch, and Reserve (AWaRe) classification. Consumption, expressed as DOT, was aggregated within each category, and the relative share of Access-, Watch-, and Reserve-group agents was determined for each study year.

### 2.4. Statistical Analysis

All data processing, statistical analysis, and visualization were performed in the R programming language version 4.4.2 [12]. Continuous variables that did not meet the assumptions of normality were summarized as medians with interquartile ranges (IQR) and compared across the four study years using the Kruskal–Wallis rank-sum test. Where the omnibus test was significant, pairwise differences were assessed using Dunn’s post hoc test with Bonferroni correction. Categorical variables and frequency distributions, such as the distribution of pathogen types and of discharge diagnoses across years, were compared using Pearson’s Chi-squared test. Monotonic trends across the ordered study years, including the proportion of antibiotic-treated patients, the proportions of individual diagnostic categories, and the viral-linked share of specific agents, were evaluated using the Cochran–Armitage test for trends. Changes in the counts of ceftriaxone use between periods were tested using the Poisson exact test. Associations between antibiotic choice and pathogen type (viral versus bacterial) were assessed using Fisher’s exact test. Where multiple comparisons were performed, *p*-values were adjusted using the Benjamini–Hochberg false discovery rate procedure. A *p*-value below 0.05 was considered statistically significant.

## 3. Results

### 3.1. Patient Characteristics and Antibiotic Exposure

Of the 567 admitted children, 382 (66.3%) were treated with antibiotics. The proportion of antibiotic-treated patients rose steadily across the study period, from 60.5% (26/43) in 2021 to 66.2% (106/160) in 2022, 69.2% (126/182) in 2023, and 71.4% (130/182) in 2024 (Table 1), but the trend did not reach statistical significance (*p* = 0.13, Cochran–Armitage test for trends).

Both patient age and length of stay (LOS) of antibiotic-treated patients were highest in 2021 and stabilized thereafter. The median age of treated patients fell from 1.6 years in 2021 to 0.4 years in 2022, remaining low in 2023 (0.8 years) and 2024 (0.4 years) (*p* = 0.066, Kruskal–Wallis test). Median LOS likewise decreased from 10 days in 2021 to 5–6 days in the subsequent periods (*p* = 0.026, Kruskal–Wallis test).

### 3.2. Aggregate Antibiotic Consumption

**Figure 1:**
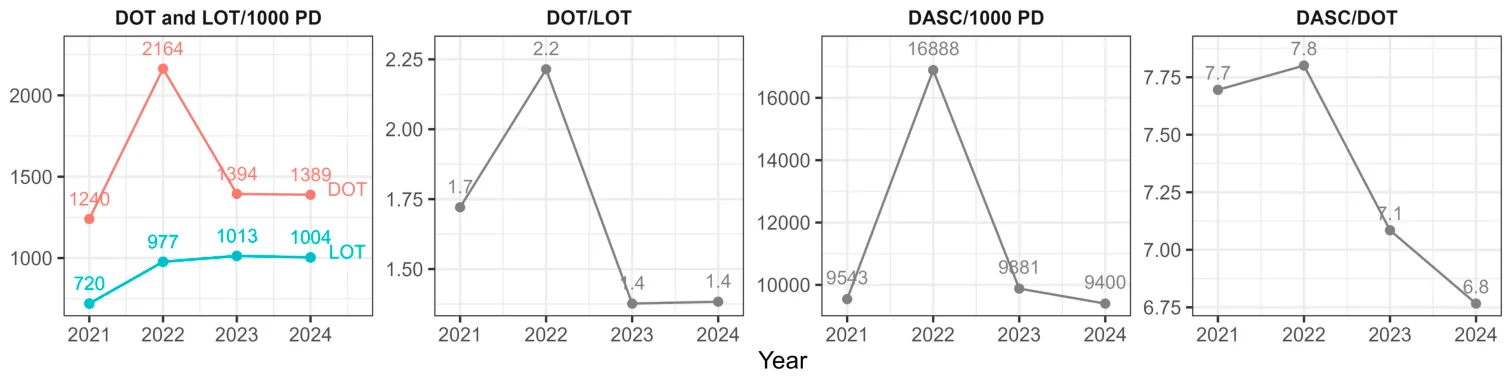
Aggregate antibiotic usage metrics over the four time periods. The numbers above the points represent the exact metric values.

Aggregate consumption metrics, expressed per 1000 patient-days (PD), are summarized in Table 2 and Figure 1.

Length of Therapy (LOT) increased from 720/1000 PD in 2021 to 977 in 2022 and then plateaued at approximately 1000/1000 PD. Days of Therapy (DOT) followed a different trajectory, peaking sharply in 2022 (2164/1000 PD) before stabilizing at approximately 1400 in 2023 and 2024. Days of Antibiotic Spectrum Coverage (DASC) mirrored this pattern, rising from 9543/1000 PD in 2021 to a maximum of 16,888 in 2022 and then falling back to similar values in 2023 and 2024 (Figure 1).

The DOT/LOT ratio peaked at 2.2 in 2022 before settling to 1.4 in both 2023 and 2024 (from 1.7 in 2021). Such a prominent peak was absent in the DASC/DOT ratio, which declined steadily in 2023 and 2024 compared to 2021 and 2022 (7.7, 7.8, 7.1, and 6.8) (Figure 1).

The difference across years was statistically significant for all the above metrics with *p*-values lower than 0.001 (Kruskal–Wallis test). The post hoc analysis (Dunn’s pairwise post hoc with Bonferroni correction) indicated that 2021 was significantly different than all subsequent years, which did not differ from one another.

### 3.3. Most Commonly Used Antibiotics and AWaRe Classification

When antibiotics were grouped according to the WHO AWaRe framework, Watch-group agents dominated consumption throughout the study period and their share increased over time, from approximately 74% in 2021 to about 87% in 2022–2024.

**Figure 2:**
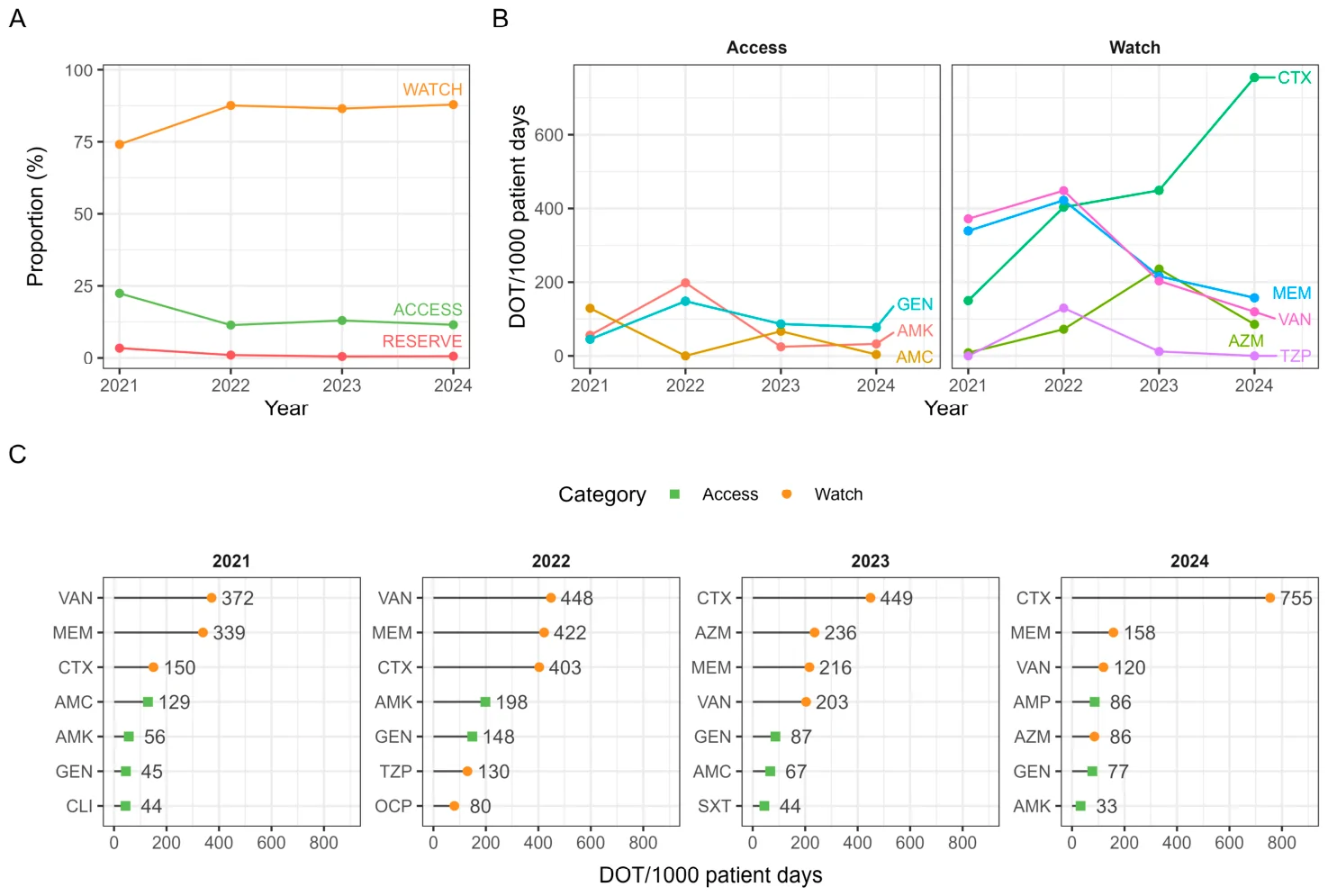
Antibiotic usage according to the AWaRe classification. (**A**) Percentage of antibiotics in each category over the years. (**B**) DOT/1000 patient-days for the eight most commonly used antibiotics. (**C**) DOT/1000 patient-days for seven most common antibiotics for each of the four time periods. Abbreviations: VAN = Vancomycin, MEM = Meropenem, CTX = Ceftriaxone, AMC = Amoxicillin/clavulanate, AMK = Amikacin, GEN = Gentamicin, CLI = Clindamycin, TZP = Piperacillin/tazobactam, OCP = Other cephalosporins, AZM = Azithromycin, SXT = Sulfamethoxazole/trimethoprim, AMP = Ampicillin.

The Access-group share fell correspondingly, from roughly 22% in 2021 to about 11–13% in the subsequent periods, while Reserve-group agents remained negligible throughout (approximately 3% in 2021, declining to about 1%) (Figure 2A).

The eight most commonly used antibiotics and their AWaRe categories are shown in Figure 2B. The most pronounced change was a steep rise in ceftriaxone use of more than fivefold between 2021 and 2024 (150 vs. 755 DOT/1000 PD) (*p* < 0.001, Poisson exact test), becoming the most-used agent in the final period (Figure 2C). The broad-spectrum Watch agents vancomycin and meropenem were the leading agents in 2021 (372 and 339 DOT/1000 PD, respectively) and 2022 (448 and 422), and remained among the most-used antibiotics in every period. Azithromycin rose to prominence from 2023 onward (236 DOT/1000 PD in 2023; 86 in 2024). Access-group agents such as amikacin, gentamicin, amoxicillin/clavulanate, and ampicillin appeared among the most-used antibiotics but at consistently lower magnitudes than the dominant Watch agents (Figure 2C).

### 3.4. Antibiotic Use with Respect to Discharge Diagnosis

**Figure 3:**
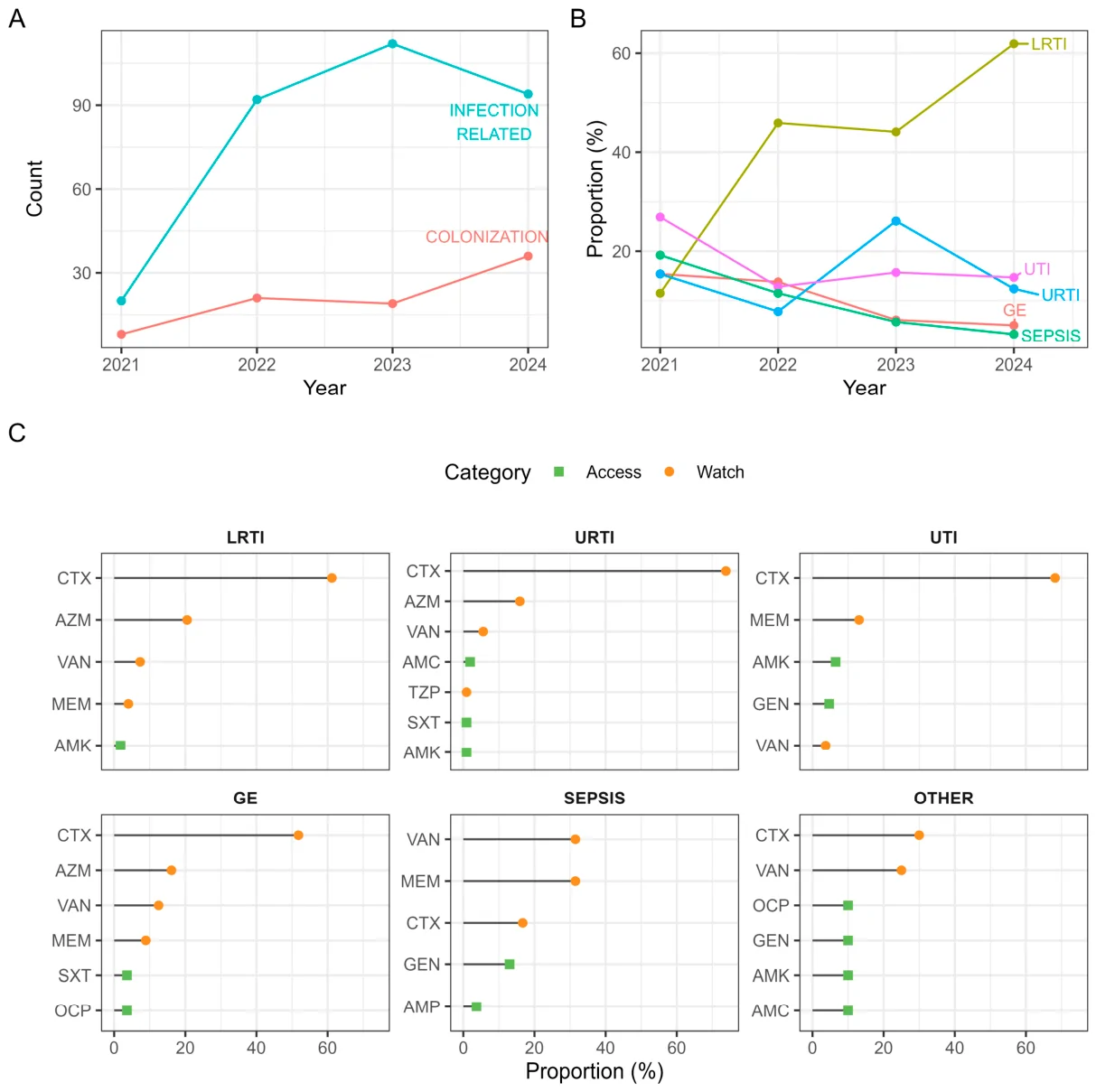
(**A**) Number of antibiotic-treated patients with and without an infection-related diagnosis. (**B**) Proportion of top 5 most common infection diagnoses over the four observed periods. (**C**) The most commonly used antibiotics for each diagnosis group. Abbreviations: VAN = Vancomycin, MEM = Meropenem, CTX = Ceftriaxone, AMC = Amoxicillin/clavulanate, AMK = Amikacin, GEN = Gentamicin, TZP = Piperacillin/tazobactam, OCP = Other cephalosporins, AZM = Azithromycin, SXT = Sulfamethoxazole/trimethoprim, AMP = Ampicillin, UTI = urinary tract infection, GE = enteritis/gastroduodenitis, URTI = upper respiratory tract infection, LRTI = lower respiratory tract infection.

The number of antibiotic-treated patients with an infection-related discharge diagnosis rose markedly from 2021 to 2023 and remained high in 2024, paralleling the overall increase in admissions, whereas the number of antibiotic-treated patients who had microbiological evidence of colonization without a concurrent infection-related discharge diagnosis remained comparatively low (Figure 3A).

The overall distribution of infection diagnoses shifted significantly across the four study years (*p* < 0.001, Pearson’s Chi-squared test) (Figure 3B). Among the leading diagnoses, the proportion attributable to lower respiratory tract infection (LRTI) increased substantially, rising from 11.5% in 2021 to 61.9% in 2024 and representing the largest diagnostic share by the final year (*p* < 0.001, Cochran–Armitage trend test). Enteritis/gastroduodenitis (GE) and sepsis each showed statistically significant decreasing trends over the same period (GE: 15.4% to 5.0%, sepsis: 19.2% to 3.2%; *p* < 0.001, Cochran–Armitage trend test). Urinary tract infections (UTI) and upper respiratory tract infection (URTI) did not exhibit a statistically significant linear trend (UTI: *p* = 0.375; URTI: *p* = 0.247, Cochran–Armitage trend test).

The most commonly used antibiotics differed by diagnosis group (Figure 3C). Ceftriaxone was the leading agent in the respiratory and gastrointestinal diagnosis groups, followed by azithromycin and vancomycin. In contrast, the bacterial-typical diagnoses were treated predominantly with broad-spectrum agents directed at bacterial pathogens: UTI was managed mainly with ceftriaxone, meropenem, and aminoglycosides (AMK, GEN), and sepsis mainly with vancomycin and meropenem, followed by ceftriaxone and gentamicin (Figure 3C).

### 3.5. Antibiotic Use with Respect to Pathogen Type

**Figure 4:**
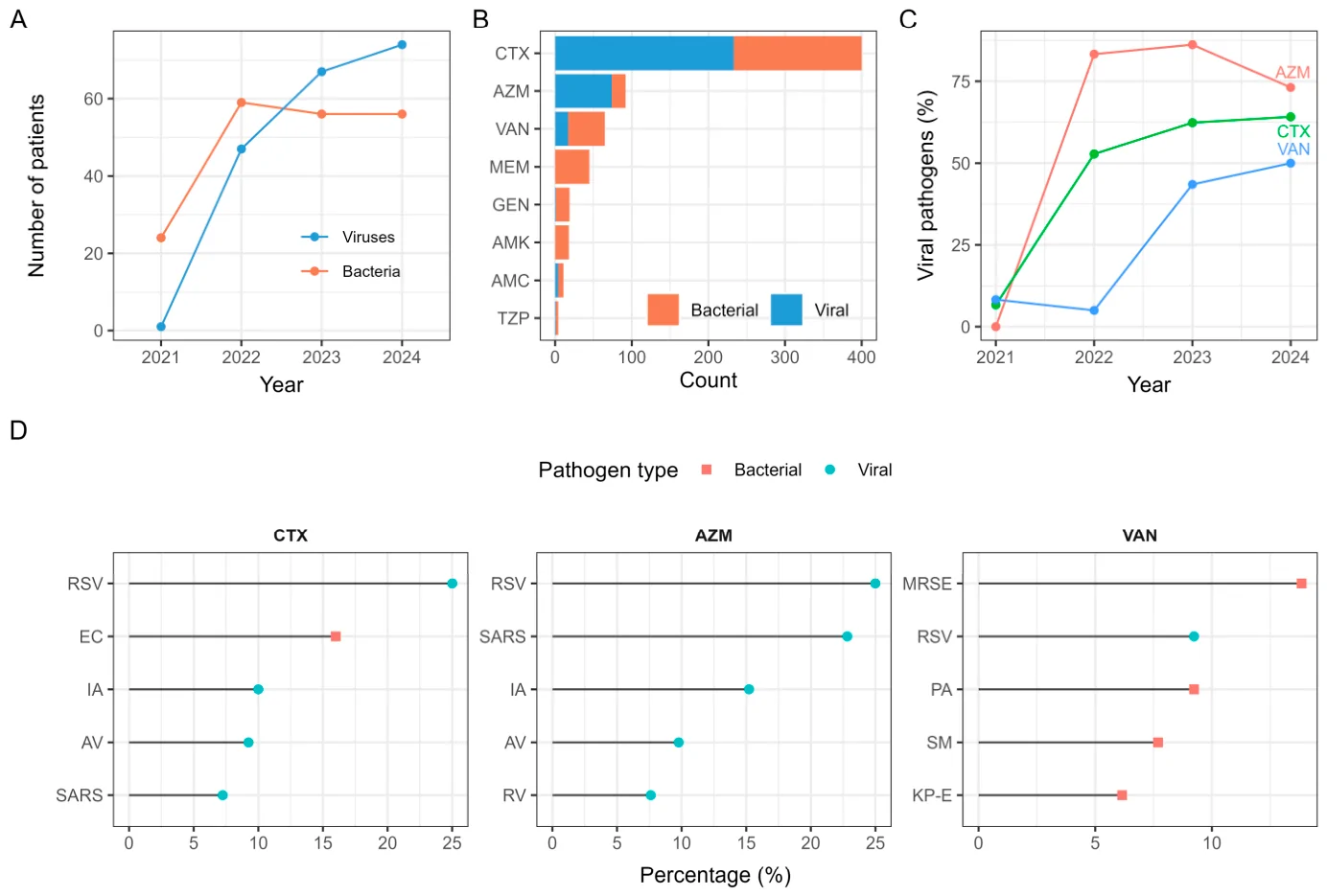
(**A**) Pathogen type trends over the observed time period. (**B**) Antibiotic prescriptions with regard to pathogen type. (**C**) Proportion of prescriptions linked to a viral pathogen for the three agents most often prescribed in viral infections. (**D**) The most common pathogens for the three antibiotics that were commonly prescribed for viral pathogens. Abbreviations: CTX = Ceftriaxone, AZM = Azithromycin, VAN = Vancomycin, MEM = Meropenem, GEN = Gentamicin, AMC = Amoxicillin/clavulanate, AMK = Amikacin, TZP = Piperacillin/tazobactam, RSV = Respiratory syncytial virus, IA = Influenza A, AV = Adenovirus, SARS = SARS-CoV-2, RV = Rotavirus, EC = *Escherichia coli*, MRSE = Methicillin-resistant *Staphylococcus epidermidis*, PA = *Pseudomonas aeruginosa*, SM = *Stenotrophomonas maltophilia*, KP-E = *Klebsiella pn.* ESBL.

To examine whether antibiotic selection corresponded to the type of infecting agent, prescriptions were linked to whether the patient’s identified pathogen was viral or bacterial (Figure 4). The distribution of pathogen types changed significantly across years (*p* < 0.001, Pearson’s Chi-squared test), with viral pathogens overtaking bacterial pathogens from 2023 onward (Figure 4A).

When individual antibiotics were stratified by underlying pathogen type, a clear discordance emerged for the most-used agents (Figure 4B). Ceftriaxone, the most frequently prescribed antibiotic overall, was administered more often to children whose identified pathogen was viral (58.2%; *p* < 0.001, Fisher’s exact test) than to those with a bacterial pathogen, and azithromycin showed the same predominantly viral pattern, with 80.4% of prescriptions occurring in children with a viral isolate (*p* < 0.001, Fisher’s exact test). By contrast, vancomycin was prescribed predominantly to children with bacterial isolates (73.8%; *p* < 0.001, Fisher’s exact test), as were gentamicin (94.7%; *p* < 0.001, Fisher’s exact test), meropenem (100% bacterial), and amikacin (100% bacterial). Amoxicillin/clavulanate was numerically more common in children with bacterial isolates (63.6% vs. 36.4%), but this difference did not reach statistical significance, likely reflecting the small number of cases (*n* = 11; *p* = 0.37, Fisher’s exact test). All *p*-values were adjusted for multiple comparisons using the Benjamini–Hochberg method.

Figure 4C shows the proportion of prescriptions linked to a viral pathogen for the three agents most often prescribed in viral infections. For ceftriaxone, the viral share rose steeply from near zero in 2021 to 52.8% in 2022 and around 60% in 2023 and 2024 (*p* < 0.001, Cochran–Armitage trend test). Azithromycin showed a consistently high viral share across all years with sufficient data (no usage in 2021; 83.3% in 2022, 86.2% in 2023, and 73.1% in 2024), reflecting stable predominance of viral-pathogen prescriptions rather than a transient peak. Vancomycin showed a low viral share in 2021 and 2022 (8.3% and 5.0%, respectively), but this increased markedly to 43.5% in 2023 and 50.0% in 2024, a trend that reached statistical significance (*p* = 0.001, Cochran–Armitage trend test).

Among children given ceftriaxone, the single most common underlying isolate was respiratory syncytial virus (RSV, approximately 25%), followed by *Escherichia coli*, influenza A, adenovirus, and SARS-CoV-2 (Figure 4D). Among azithromycin recipients, the leading isolates were likewise viral, principally RSV and SARS-CoV-2, followed by influenza A and adenovirus. In contrast, vancomycin recipients were characterized predominantly by bacterial isolates such as Methicillin-resistant *Staphylococcus epidermidis* and *Pseudomonas aeruginosa*. Taken together, these results indicate that the rising, Watch-dominated antibiotic consumption was increasingly directed at children whose identified infection was viral, with ceftriaxone use in RSV-positive children the most prominent example.

## 4. Discussion

This study characterized antimicrobial consumption and prescribing patterns in children hospitalized with a positive microbiological finding during and after the COVID-19 pandemic. The proportion of admitted children receiving antibiotics increased from 60.5% in 2021 to 71.4% in 2024 (Table 1). On its own this rise might suggest an increasing bacterial burden, but bacterial pathogens predominated only in 2021, after which viral pathogens took over and the patient population became markedly younger as restrictions eased and infants were exposed to a resurgent pool of respiratory viruses [11]. These findings are consistent with the well-documented tendency to treat hospitalized children with viral respiratory illness empirically [3,4]. The shift in the present cohort toward young infants with severe-appearing RSV and influenza disease plausibly lowered the threshold for empirical therapy. This pattern was most evident with ceftriaxone, whose use rose more than fivefold over the study period and which was administered increasingly to children whose only identified pathogen was viral, most often RSV. The probable overuse of ceftriaxone is concerning as it confers little benefit to the treated child while contributing to the selection of resistant Gram-negative organisms such as *Escherichia coli* and *Klebsiella pneumoniae* that are already among the most threatening pathogens in pediatric practice [13].

The aggregate consumption metrics added temporal nuance (Figure 1). The 2022 peak in DOT/LOT ratio (2.2 concurrent agents per therapy-day) and DASC coincided with the wave of COVID-19 infections in the first quarter of 2022, and is most readily explained by broad, multi-agent empirical regimens administered during that period. This mirrors international experience in COVID-19 cohorts, in which Watch-group antibiotics were the most prescribed across successive waves despite a low incidence of bacterial co-infection [14]. The DASC/DOT ratio—the mean spectrum breadth per antibiotic-day—declined steadily across the four periods, suggesting a modest de-escalation in per-day spectrum even as the number of children exposed to antibiotics grew. The value of reporting DASC alongside DOT is evident here, because days of therapy alone cannot capture stewardship efforts that favor narrow-spectrum agents.

Regarding the AWaRe analysis, Watch-group agent use was around 80%, while the Access share was approximately 15–20% and Reserve agents remained negligible (Figure 2A). The Access proportion therefore remained well below the WHO benchmark of at least 60% of consumption, with a target of 70% of prescriptions globally by 2030 throughout the study. This degree of Watch-group predominance is typical of hospital settings before, during and after the pandemic, with ceftriaxone the most common agent in young children [15,16,17,18]. The dominance and continued rise in ceftriaxone in the present cohort (Figure 2C) is thus consistent with a broad international signal rather than a local occurrence [15,17,18].

The interpretation of these AWaRe proportions warrants caution, because the framework’s targets and its very structure remain contested. The global Access targets (the ≥60% consumption benchmark and the 70% prescription target) have been argued to lack epidemiological justification and may not fully capture regional disease patterns, such that a sub-benchmark Access share may partly reflect local case-mix rather than poor stewardship [19]. More fundamentally, the Access/Watch dichotomy is a relatively blunt proxy for prescribing quality, as category membership is fixed at the level of the molecule and does not in itself encode whether a given agent was appropriate for the specific indication. As Hsia et al. noted in the first multinational pediatric application of the classification, improving prescribing is more complex than simply increasing prescriptions for Access antibiotics, and a high Watch share cannot be equated with inappropriate use [15]. Additionally, drug-level inconsistencies distort the metric independently of stewardship. For example, trimethoprim-sulfamethoxazole is classified as Access despite a broad spectrum and frequent long-term prophylactic use (e.g., in people living with HIV), inflating the Access percentage without any gain in stewardship. Furthermore, doxycycline (Access) and minocycline (Watch) have near-identical spectra, so substituting one for the other changes the metric but not the clinical reality [19]. Epidemic conditions add to the issue, as during a *Mycoplasma pneumoniae* epidemic in Tokyo the Access proportion reportedly fell from 64% to 48% because first-line macrolides are classified as Watch [20].

Azithromycin use illustrates this second mechanism. A macrolide and therefore a Watch agent, it was used widely in patients with COVID-19, particularly in combination with hydroxychloroquine, on the basis that its immunomodulatory and antiviral properties might benefit severe disease [21]. This explains the spike in azithromycin use in 2022 (Figure 4C), and signals that Watch-group predominance in our cohort should therefore be read as a structural and epidemiological signal as much as a stewardship one, not a straightforward indictment of local prescribing.

### Limitations

When interpreting the absolute values of the above metrics, it is important to note that the study was restricted to the first quarter of each year and to a single tertiary center, which limits generalizability and precludes assessment of seasonal variation across the full year. Because pediatric antibiotic prescribing peaks in the cold season and drops sharply in summer, with approximately a fourfold difference [22,23], the consumption magnitudes reported here should be regarded as reflecting a seasonal maximum rather than an annual average. Furthermore, the inspected cohort is a subpopulation of hospitalized pediatric patients with a positive microbiological finding, including patients from the pediatric intensive care unit. Consequently, it is likely that a proportion of these children were critically ill and that broad empirical cover was more defensible in those cases than in a general ward population. The cohort was also very young (median age 0.4–0.8 years in 2022–2024, with approximately 80% of patients under 2 years), and prescribing rates are known to be strongly age-dependent, with studies reporting a near-doubling in the youngest age group [24,25]. Diagnostic uncertainty in preverbal infants, parental expectations, and concern over rapid clinical deterioration all plausibly contribute to this effect. Age and case-mix therefore inflate the observed rates relative to a general pediatric population.

Moreover, the identification of a viral pathogen does not by itself exclude clinical justification of antibiotic prescription, so some broad-spectrum prescribing in RSV- or influenza-positive children may have been clinically justified, for example for suspected secondary bacterial pneumonia or for sepsis rule-out in young infants in whom clinical deterioration is difficult to predict. The data therefore describes antibiotics prescribed to children whose identified pathogen was viral, rather than definitively unnecessary treatment, and culture-negative co-infection cannot be entirely excluded. Finally, prescribing appropriateness was inferred from pathogen and diagnosis data rather than from a chart-level adjudication of indication. Future work incorporating severity scores, inflammatory markers, and explicit indication review across multiple centers and all four quarters would allow the extent of genuinely avoidable antibiotic use to be quantified more precisely.

## 5. Conclusions

In hospitalized children spanning the transition from the COVID-19 pandemic to the post-pandemic period, antibiotic exposure increased and remained dominated by Watch-group agents, with ceftriaxone rising to become the predominant antibiotic. Linking prescriptions to the underlying pathogen revealed that this broad-spectrum use was directed increasingly at children with viral infections, RSV foremost among them, despite a concurrent shift in the case-mix away from bacterial disease documented in the companion analysis. These pathogen-aware findings identify a high-yield and actionable antimicrobial stewardship target and illustrate the value of combining quantitative and qualitative consumption metrics (DOT, LOT, DASC, and the AWaRe framework) with pathogen-level data to guide prescribing in the evolving post-pandemic landscape.

## Figures and Tables

**Table 1:** Patient characteristics over the four observed periods. The aggregate statistics are presented as medians (IQR).

Feature/Year	2021	2022	2023	2024	*p*-Value
Admitted	43	160	182	182	<0.001
Antibiotic-treated	26 (60.5%)	106 (66.2%)	126 (69.2%)	130 (71.4%)	0.13
Age	1.6 (0.4–7.2)	0.4 (0.1–3.1)	0.8 (0.3–4.8)	0.4 (0.1–2.9)	0.066
LOS	10 (4–21.5)	5 (3–9.5)	5 (3–8)	6 (4–8)	0.026

**Table 2:** Antibiotic usage metrics over the four time periods.

Metric/Year	2021	2022	2023	2024	*p*-Value
LOT/1000 PD	720	977	1013	1004	<0.001
DOT/1000 PD	1240	2164	1394	1389	<0.001
DASC/1000 PD	9543	16,888	9881	9400	<0.001
DOT/LOT	1.7	2.2	1.4	1.4	<0.001
DASC/DOT	7.7	7.8	7.1	6.8	<0.001

## Data Availability

Dataset available upon reasonable request from the authors.

## Abbreviations

The following abbreviations are used in this manuscript:

DOT	Days of Therapy
LOT	Length of Therapy
DASC	Days of Antibiotic Spectrum Coverage
PD	Patient Days
AWaRe	Access/Watch/Reserve
AMR	Antimicrobial Resistance
WHO	World Health Organization
URTI	Upper Respiratory Tract Infection
LRTI	Lower Respiratory Tract Infection
UTI	Urinary Tract Infection
GE	Enteritis/gastroduodenitis
CTX	Ceftriaxone
VAN	Vancomycin
MEM	Meropenem
AZM	Azithromycin
AMC	Amoxicillin/clavulanate
AMK	Amikacin
AMP	Ampicillin
GEN	Gentamicin
CLI	Clindamycin
TZP	Piperacillin/tazobactam
OCP	Other cephalosporins
SXT	Sulfamethoxazole/trimethoprim
RSV	Respiratory Syncytial Virus
IA	Influenza A
RV	Rotavirus
SARS	SARS-CoV-2
AV	Adenovirus
MRSE	Methicillin-resistant *Staphylococcus epidermidis*
PA	*Pseudomonas aeruginosa*
SM	*Stenotrophomonas maltophilia*
KP-E	*Klebsiella pn.* ESBL
